# LigaSure Vessel Sealing System for Small Bowel Transection During Roux Limb Construction

**DOI:** 10.7759/cureus.21287

**Published:** 2022-01-16

**Authors:** Srikanth Gadiyaram, Murugappan Nachiappan

**Affiliations:** 1 Department of Surgical Gastroenterology and Minimally Invasive Surgery, Sahasra Hospitals, Jayanagar, Bangalore, IND

**Keywords:** roux-en-y reconstruction, transection, small bowel, roux limb, ligasure

## Abstract

Roux limb construction is an essential part of several major reconstructive hepatobiliary and upper gastrointestinal surgeries. This can be achieved with a stapling device or suturing. For over two decades, the LigaSure vessel sealing systems (Medtronic, MN, USA) have been in use for omental division, mesenteric transection, and sealing of vessels. We used the LigaSure vessel sealing system with a ForceTriad energy platform (Medtronic) for transection of the bowel during the formation of the Roux limb for a Roux-en-Y reconstruction. Between July 2019 and December 2020, patients who had Roux limb construction as part of a pancreato-enteric anastomosis in surgery for chronic pancreatitis were analysed. The data was reviewed from a prospectively maintained database. Fifteen patients had undergone surgery for chronic pancreatitis. The mentioned technique takes approximately eight minutes to construct a Roux limb. There was no bleeding from the gut ends that had been transected. There was no breach in the bowel’s seal. The field was free of enteric contamination. In the post-operative course of these individuals, there was no Roux limb-related morbidity. This procedure is useful because it is cost-effective, time-saving, dependable, and prevents contamination and blood loss. It is also simple to learn and apply.

## Introduction

Several important reconstructive hepatobiliary and upper gastrointestinal operations need Roux limb creation. The jejunal transection during Roux limb construction is done by suturing or stapling techniques. Suture closure of the transected ends is associated with possible contamination of the operative field and a longer time to obtain appropriate mucosal inversion. The use of stapling technique achieves secure and quick transection but comes at an additional cost.

LigaSure (Medtronic, MN, USA) seals tissues with a combination of energy and mechanical compression. Collagen and elastin are denaturized as a consequence of this. A hemostatic and leak-resistant tissue plug is generated as a result [1]. The output of the generating machine is influenced by the impedance of the tissue in the instrument [2]. LigaSure is used to seal vessels with a diameter of up to 7 mm.

We applied the LigaSure vessel sealing system with a ForceTriad energy platform (Medtronic) for the transection of the bowel during the construction of the Roux limb for a Roux-en-Y reconstruction. We analysed patients who underwent Roux limb construction as a part of pancreato-enteric anastomosis in surgery for chronic pancreatitis over one and a half years from July 2019 to December 2020. We reviewed data from a prospectively maintained database. Fifteen patients had undergone surgery for chronic pancreatitis. Informed consent was obtained from all patients. The described technique pertains to the transection of bowel by a bipolar energy device in place of a monopolar cautery and a reinforcing suture layer of the transected bowel, similar to a sutured closure. It is important to note that this study does not evaluate the independent ability of LigaSure to seal the bowel.

## Technical report

**Figure 1:**
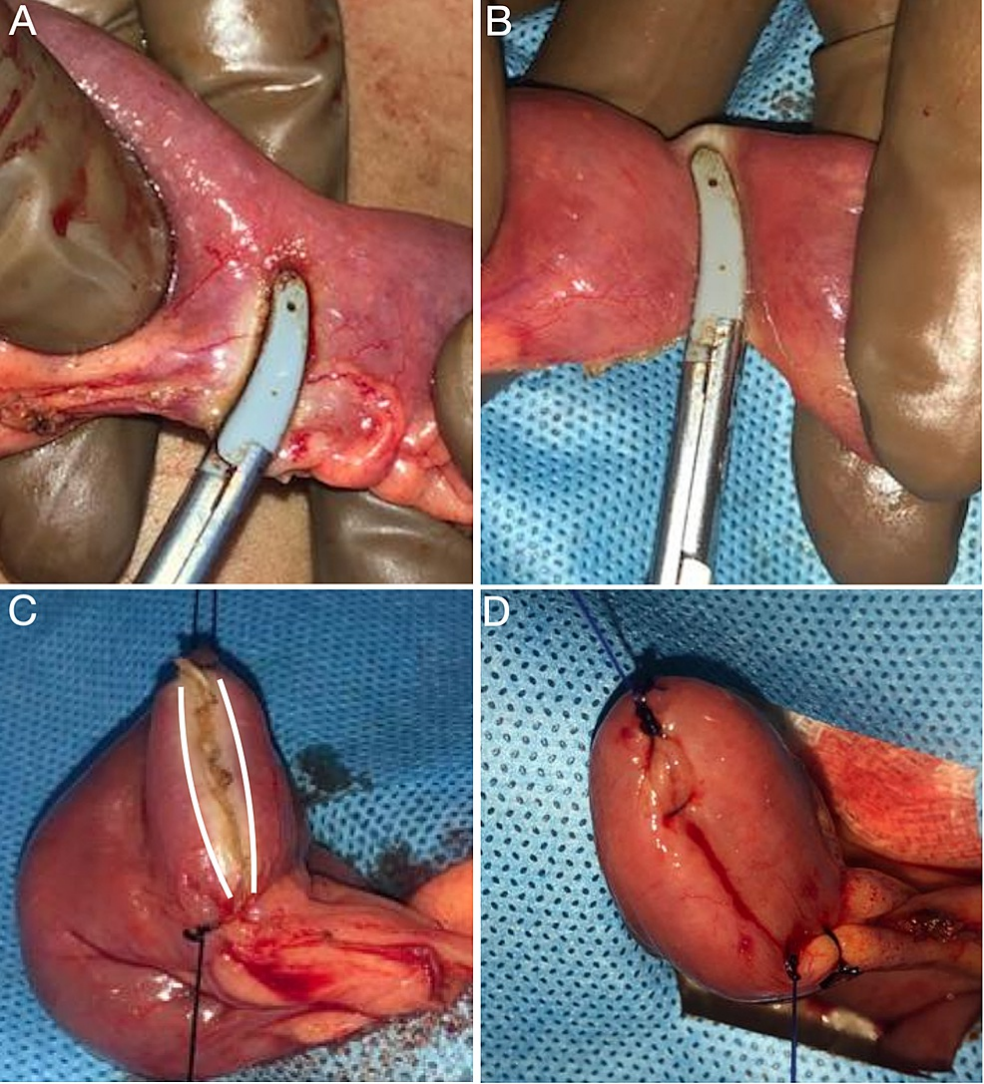
Operative photographs (A) Application of LigaSure for bowel transaction after division of the mesentery, (B) completion of bowel transection, (C) transected sealed bowel end held with stay sutures; note the zone of thermal spread, (D) completed seromuscular polypropylene continuous suture to cover the sealed end.

**Figure 2:**
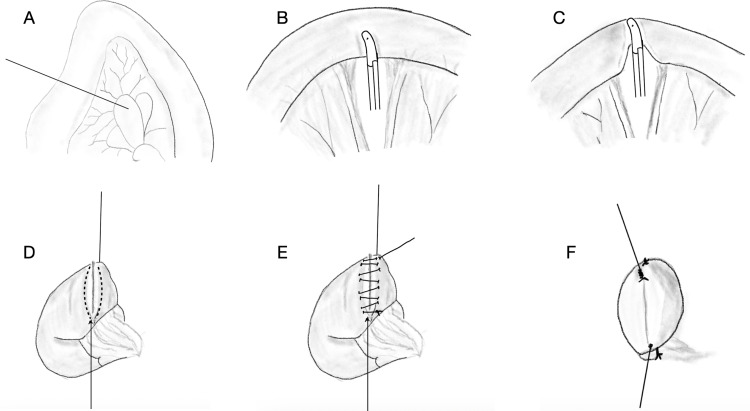
Illustrations demonstrating the use of LigaSure for bowel transection (A) Identification of a suitable point of jejunum for transection, (B) application of LigaSure for bowel transection after mesenteric division, (C) completion of transection, (D) sealed bowel end prepared for inverting seromuscular layer of polypropylene sutures, (E) polypropylene continuous suture layer taken away from the zone of possible thermal spread (shown by dashed lines), (F) completed seromuscular layer.

The jejunal mesenteric arcade was evaluated under transillumination after verifying that the mesentery was enough to allow the Roux limb to reach its destination without tension. An appropriate site of the jejunum, approximately 15-20 cm from the ligament of Treitz, was selected for transection. A LigaSure small jaw was employed for the mesenteric division. The ForceTriad energy platform's energy setting was set to two bars. After dividing the mesentery, two serial applications of the LigaSure small jaw were used to transect the gut from the mesenteric to the anti-mesenteric end. The device was used to first seal the intestine and then divide it with the inbuilt cutting mechanism after each use. The transected ends of the intestine were then overrun with polypropylene 3-0 continuous sutures, starting at the mesenteric end and extending to the anti-mesenteric end, reinforcing the cut ends. The bites were taken while staying away from the area of thermal spread (2 mm). This totally inverts the bowel's sealed boundaries while also lowering the seal's tension. The technique is shown by operative images in Figures 1A-1D and by illustrations in Figures 2A-2F, respectively. The side-to-side jejunojejunostomy (functional end-to-side) was accomplished by a single-layer continuous 3-0 polydioxanone suture (PDS), to complete the Roux limb.

The mentioned technique took eight minutes on average to construct a Roux limb. There was no bleeding from the transected bowel ends, and the intestinal seal had not been breached in any of the patients. We were able to transect the bowel without contaminating the field with enteric contents. In the post-operative course of these individuals, there was no Roux limb-related morbidity.

## Discussion

In the last two decades, LigaSure vessel sealing systems have been used in a wide range of specialties [3-6]. LigaSure has largely been used to seal vessels up to 7 mm in diameter. In animal studies, attempts to plug cystic ducts and lung parenchyma have been made [6,7].

The suggested LigaSure small jaw bowel transection method has various advantages over previous approaches. It achieves a reliable seal, which prevents enteric contamination of the field. Prior to being reinforced with a seromuscular continuous suture, none of our patients had a burst. There was no blood loss from the transected bowel's cut edges. In comparison to other methods, this one requires no changes to the instrumentation, which saves time. The approach is also simple to teach and replicate. Finally, because LigaSure is commonly utilised for dissection and vascular sealing in major hepatobiliary or upper gastrointestinal procedures, there is no additional cost for bowel transection.

Inconclusive results have been obtained from ex vivo animal studies on intestinal transection with LigaSure. According to some studies, the burst pressure is lower than that achieved with a stapler or a hand-sutured bowel closure [2,8]. Salameh et al. discovered that the most common location of seal give-away is in the middle, and they speculated that this could be due to the application of the small jaw twice. For a single application, they used the LigaSure™ Xtd Hand Switching Instrument with its long jaw. Despite this, they discovered that stapler burst pressures were substantially higher than LigaSure. As a result, they came to the conclusion that LigaSure should not be used for bowel closure [8]. Others, on the other hand, have discovered that LigaSure is an effective instrument for closing the intestines when a serosa-to-serosa closure is accomplished [9].

In contrast, an in vivo animal study comparing energy sealing devices to staplers for caecal sealing discovered that energy sealing devices have a considerable increase in sealing pressure on the seventh post-operative day [10]. When compared to staplers, sealing devices caused much less ischemia. Others have gone one step further and used LigaSure in vivo experimental investigations to effectively achieve intestinal anastomosis [11].

All of these experimental approaches are different from the technique currently being explained. It features a polypropylene suture reinforcement layer. This lowers the seal's tension and inverts it, providing a second layer of protection against the leak.

In patients with an oedematous gut, a foreshortened mesentery, or a very thick mesentery as a result of inflammation, one may find it difficult to use the small jaw. It may also be challenging in patients with extrahepatic portal venous obstruction and portal hypertension, which is common in chronic pancreatitis patients. In such cases, caution should be exercised, and other intestinal transection techniques should be considered. In the uncommon case that the seal breaks during seromuscular bowel suturing, standard bowel closure techniques may be required.

## Conclusions

The use of this approach for bowel transection during reconstructive hepatobiliary and upper gastrointestinal surgeries allows for a seamless procedure. As a result, the surgical team may concentrate on the more critical parts. We believe that this procedure is useful since it is cost-effective, saves time, is dependable, and prevents contamination and blood loss. It is also simple to learn and apply.
